# An artificial intelligence model for embryo selection in preimplantation DNA methylation screening in assisted reproductive technology

**DOI:** 10.52601/bpr.2023.230035

**Published:** 2023-12-31

**Authors:** Jianhong Zhan, Chuangqi Chen, Na Zhang, Shuhuai Zhong, Jiaming Wang, Jinzhou Hu, Jiang Liu

**Affiliations:** 1 Institute of Biophysics, Chinese Academy of Science, Beijing 100101, China; 2 Guangdong Women's and Children's Hospital, Guangzhou 511400, China; 3 Beijing Obstetrics and Gynecology Hospital, Capital Medical University, Beijing Maternal and Child Health Care Hospital, Beijing 100026, China; 4 Dongguan People’s Hospital, Dongguan 523059, China; 5 University of the Chinese Academy of Science, Beijing 101408, China; 6 School of Future Technology, University of the Chinese Academy of Science, Beijing 100049, China

**Keywords:** Assisted reproductive technology, DNA methylation, Artificial intelligence, Embryo selection

## Abstract

Embryo quality is a critical determinant of clinical outcomes in assisted reproductive technology (ART). A recent clinical trial investigating preimplantation DNA methylation screening (PIMS) revealed that whole genome DNA methylation level is a novel biomarker for assessing ART embryo quality. Here, we reinforced and estimated the clinical efficacy of PIMS. We introduce PIMS-AI, an innovative artificial intelligence (AI) based model, to predict the probability of an embryo producing live birth and subsequently assist ART embryo selection. Our model demonstrated robust performance, achieving an area under the curve (AUC) of 0.90 in cross-validation and 0.80 in independent testing. In simulated embryo selection, PIMS-AI attained an accuracy of 81% in identifying viable embryos for patients. Notably, PIMS-AI offers significant advantages over conventional preimplantation genetic testing for aneuploidy (PGT-A), including enhanced embryo discriminability and the potential to benefit a broader patient population. In conclusion, our approach holds substantial promise for clinical application and has the potential to significantly improve the ART success rate.

## INTRODUCTION

The application of ART is increasing rapidly, with more than three million ART cycles performed globally and over one million in China annually in recent years (Chambers *et al.* 2021; Qiao *et al.* 2021). Despite the high demand, the global success rate of ART remains at approximately 30% (De Geyter *et al.* 2018; Qiao *et al.* 2021). Research has linked embryo genetic factors to ART outcomes, leading to the development of preimplantation genetic screening (PGS) (Graham *et al.* 2023; Handyside *et al.* 1990). However, the overall live birth rate has no significant improvement after screening for aneuploidy, which is the most common genetic cause of pregnancy failure (Franasiak *et al.* 2014; Munné *et al.* 2019; Yan *et al.* 2021). This limitation is associated with the varied risk of aneuploid embryo production across women with different ages, highlighting the need for more effective clinical approaches. (Munné *et al.* 2019; Niederberger *et al.* 2018).

Human early embryo development experienced a dramatic global DNA methylation reprogramming (Li *et al.* 2018; Zhu *et al.* 2018). Recent clinical studies on PIMS have reported a large portion of ART embryos exhibit abnormal whole genome DNA methylation levels, which is related to significantly reduced live birth rate (Gao *et al.* 2023; Li *et al.* 2017). However, whether PIMS could be reinforced by modeling DNA methylation signatures remains as a compelling question, especially considering the high frequency of methylation errors in human preimplantation embryos (Chen *et al.* 2010; White *et al.* 2015).

AI has been increasingly leveraged in computational biology, demonstrating its effectiveness in handling large and complex biomedical data (Xu and Jackson 2019). Recent studies have introduced AI models for ART embryo screening, but their accuracies need to be further improved (Buldo-Licciardi *et al.* 2023; Diakiw *et al.* 2022; Raimundo and Cabrita 2021). Meanwhile, DNA methylation-based predictors have been developed for solving various health-related problems (Horvath and Raj 2018; McCartney *et al.* 2018; Yousefi *et al.* 2022). Their high sensitivity and accuracy indicate a promising potential for DNA methylation-based models in clinical utility (Liang *et al.* 2021; Zhou *et al.* 2022). Unfortunately, an AI model that employs DNA methylation signatures to aid ART embryo selection is yet to be established.

In this study, we proposed PIMS-AI, an AI-based model designed to select embryo for ART patients by evaluating embryos DNA methylation profiles. PIMS-AI integrates a linear transformation of whole genome DNA methylation level with a decision-tree approach based on DNA methylation signatures. The model achieved an AUC of 0.90 in cross-validation and 0.80 in independent testing. In simulated embryo selection, PIMS-AI achieved an accuracy of 81%. Taken together, these findings suggest PIMS-AI significantly outperforms PGT-A and has a great potential for future clinical application.

## RESULT

### Whole genome DNA methylation level-based outcome prediction by a linear transformation

**Figure 1:**
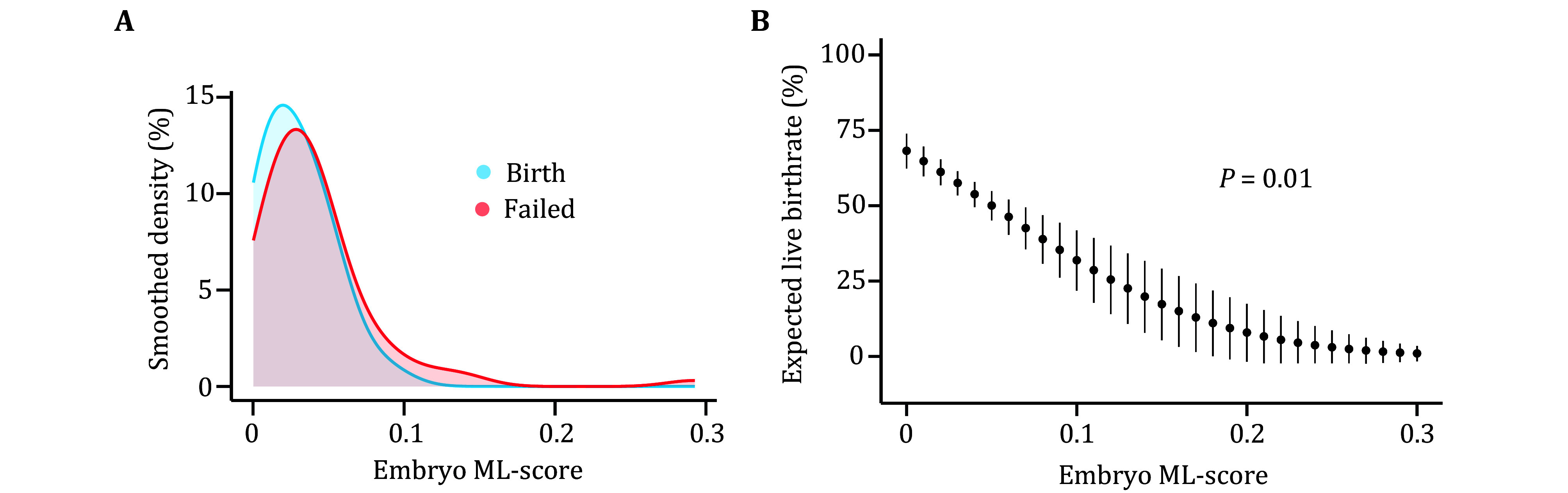
Linear relationship between embryo whole genome DNA methylation level and clinical outcome. **A** ML-score distribution of embryos with different clinical outcomes. Embryos that resulted in pregnancy failure or pregnancy loss were combined as “Failed”. **B** Expected live birth rate and standard errors relative to ML-score. The result is fitted by a binomial generalized linear model, and presented discretely with an interval of 0.01 on the *x*-axis. Linear relationship significance *P* value indicated above

A recent study reported the significant impact of whole genome DNA methylation levels on ART clinical outcome, identifying an optimal methylation level of 0.26 for the highest live birth rate (Gao *et al.* 2023). Based on this observation, we proposed a linear function to convert the whole genome DNA methylation level into the expected live birth rate. We first calculate the methylation level score (ML-score) for each embryo, which measures the absolute deviation from embryo whole genome DNA methylation level to 0.26. Consistent with our previous finding, embryos with different clinical outcomes have significantly different distributions in ML-score (Fig. 1A). A binomial generalized linear model (GLM) was then applied to quantify the relationship between ML-score and clinical outcome of embryos (Fig. 1B). Considering the confidence interval of the GLM, we anticipated that 0.03 is an optimal ML-score difference threshold for calling significant difference in embryo quality (Fig. 1B). When two embryos have ML-score difference greater than this threshold, we could confidently conclude that the embryo with lower ML-score is of superior quality than the other one. This allows a simple yet efficient embryo discrimination in the next.

### Linear model leads to significantly improved clinical outcome

**Figure 2:**
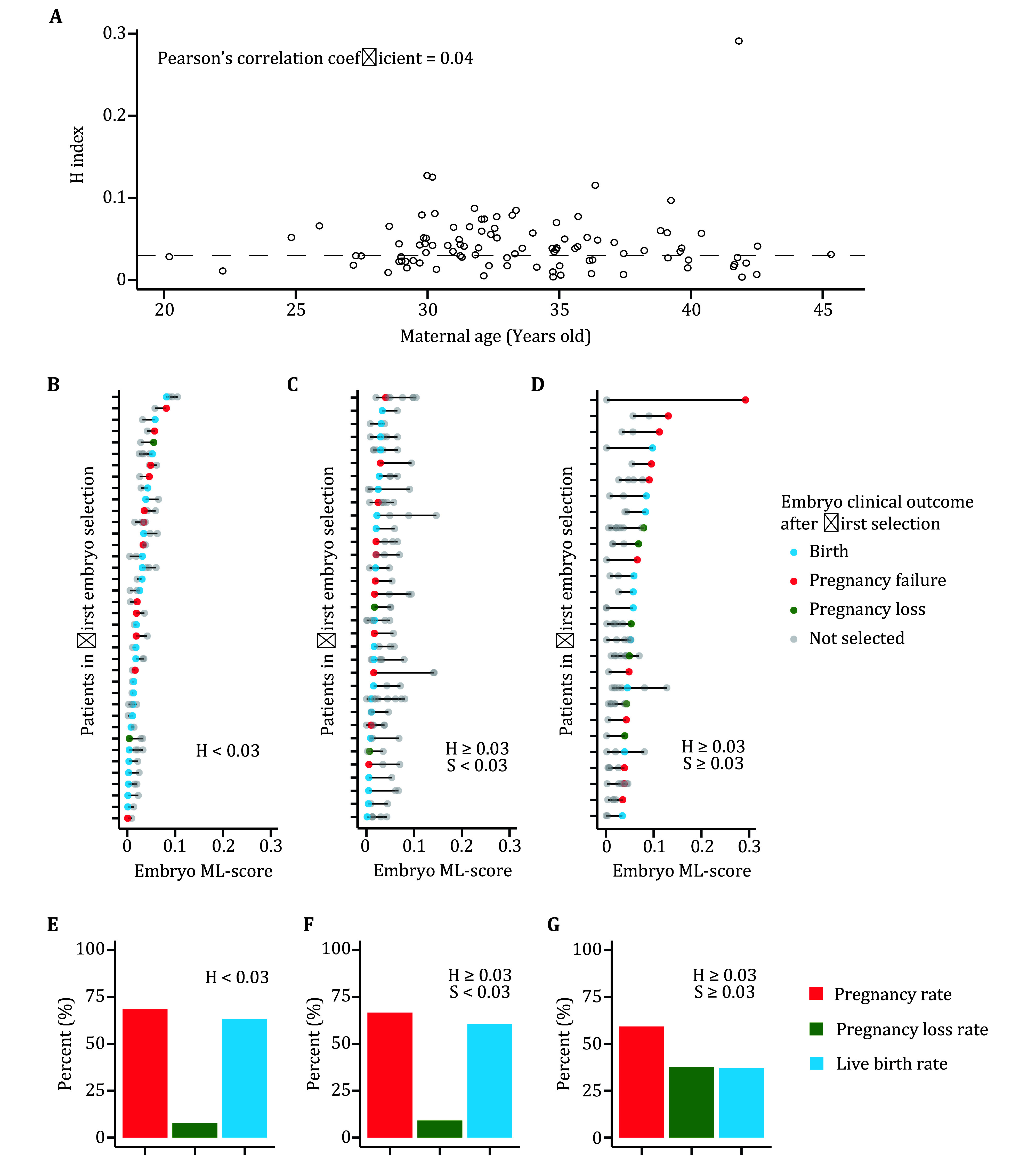
Clinical efficacy evaluation of linear model. **A** H-index distribution in patients with different maternal ages. The dashed horizontal line indicates the anticipated threshold as 0.03. **B**–**D** Clinical outcome of embryos from patients that had embryos with similar quality (**B**), selected superior embryo (**C**) and selected inferior embryo (**D**). **E**–**G** Summarized clinical outcome of patients that had embryos with similar quality (**E**), selected superior embryo (**F**) and selected inferior embryo (**G**). The inclusion criteria for each group are indicated in each corresponding panel

To assess the clinical efficacy of our linear model, we retrospectively analyzed patient embryo selections from the PIMS clinical trial. Firstly, we introduced the index of embryo heterogeneity (H-index), which measures the maximum ML-score difference among available embryos from a given patient. Based on the anticipated threshold, an H-index greater than 0.03 represents the corresponding patient having an embryo with a significant quality difference. Our findings suggest that the H-index is not related to maternal age, a high H-index value (greater than 0.03) is prevalent regardless of patient age (Fig. 2A). This suggests our linear model could potentially benefit more patients than PGT-A, which primarily helps patients with advanced maternal age (Niederberger *et al.* 2018).

We then evaluated these selections using our linear model. We proposed the index of selection status (S-index) to evaluate whether a superior or inferior embryo was selected. S-index measures the difference between the ML-score of the selected embryo and the lowest ML-score in all available embryos (including the selected one) from a given patient. Obviously, an S-index greater than 0.03 represents that the corresponding patient had selected an inferior embryo to transfer.

Based on H-index and S-index, patients were categorized into three groups. Around 38% (95% confidence interval of exact binomial test: 29% to 49%) of patients had embryos with no significant quality difference (Fig. 2B). In the rest of patients that had significant embryo heterogeneity, around half selected a superior embryo (Fig. 2C) and another half selected an inferior one (Fig. 2D). When summarizing and comparing the clinical outcome of different group, we found the first and second group have similar result (Figs. 2E and 2F). Surprisingly, selection of superior embryo significantly improved pregnancy loss rate and live birth rate compared to selection of inferior embryo (one-sided Fisher’s exact test, *P* = 0.043 and 0.059, respectively) (Figs. 2F and 2G). Meanwhile, we found maternal age has no influence on these different outcomes (mean maternal age of superior selection group = 33.77; mean maternal age of inferior selection group = 33.59; two-sided *t*-test, *P* = 0.86).

We anticipated that a significant improvement in clinical outcome could be achieved for the inferior selection group, if they had been assisted by our PIMS linear model. Many of them have unused embryos with low ML-scores (Fig. 2D). And a lower ML-score is related to a higher live-birth rate (Fig. 1B). Taken together, these results indicate PIMS could notably improve ART clinical outcomes through this simple linear model.

### Insights into epimutations related to pregnancy failure

**Figure 3:**
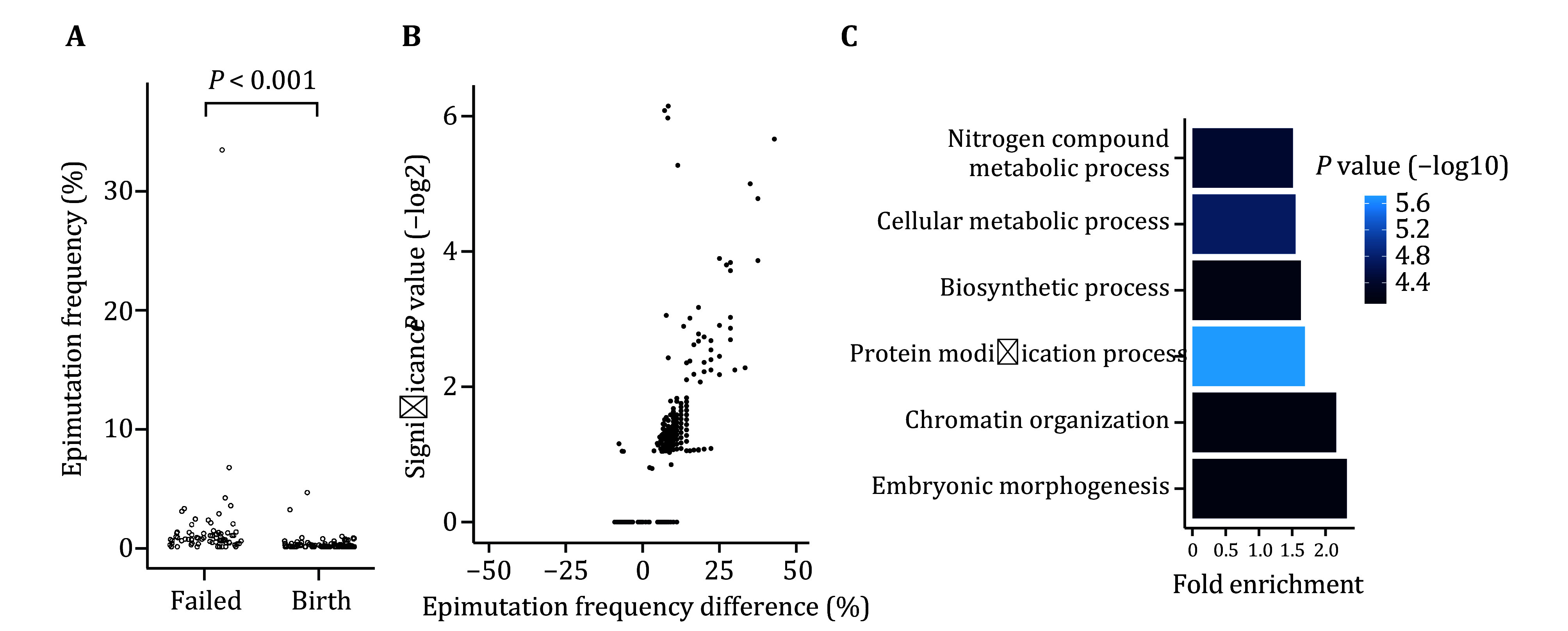
Insights into epimutations related to pregnancy failure. **A** Epimutation frequencies in embryos with different clinical outcomes. Embryos that resulted in pregnancy failure or pregnancy loss were combined as ‘Failed’. *P* value indicates the significance of the two-sided Wilcox test. **B** Epimutation frequency differences in analyzed promoters. Differences are calculated by failed embryos subtracting birth embryos. *P* value on the *Y*-axis indicated the significance of the two-sided Fisher’s exact test. **C** Gene ontology analysis result of 446 epimutation related genes. Fold enrichment indicates the gene number ratio of observed relative to expected in corresponding pathways. *P* value indicates binomial statistical significance of gene enrichment

Next, we investigated whether embryos with different clinical outcomes have differences in methylation signatures. We focused on promoters here, as they are known to have DNA methylation mutations linked with a broad variety of diseases (Ehrlich 2019; Li and Zhang 2014). We found a significantly higher epimutation frequency in embryos that failed in producing live birth (Fig. 3A). Meanwhile, we found embryo epimutation frequencies are independent from patient age (Pearson’s correlation coefficient = –0.06, *P* = 0.41). This suggests that screening embryo epimutation could potentially help patients of all ages. Furthermore, 446 promoters showed higher epimutation frequencies in failed embryos (Fig. 3B). Gene ontology analyses suggest these genes are enriched in pathways related to embryonic morphogenesis, chromatin organization and metabolism (Fig. 3C). These results provide clues for further modeling, as well as biological insights on epimutations related with pregnancy failure.

### Enhanced classification performance by a mixed model

**Figure 4:**
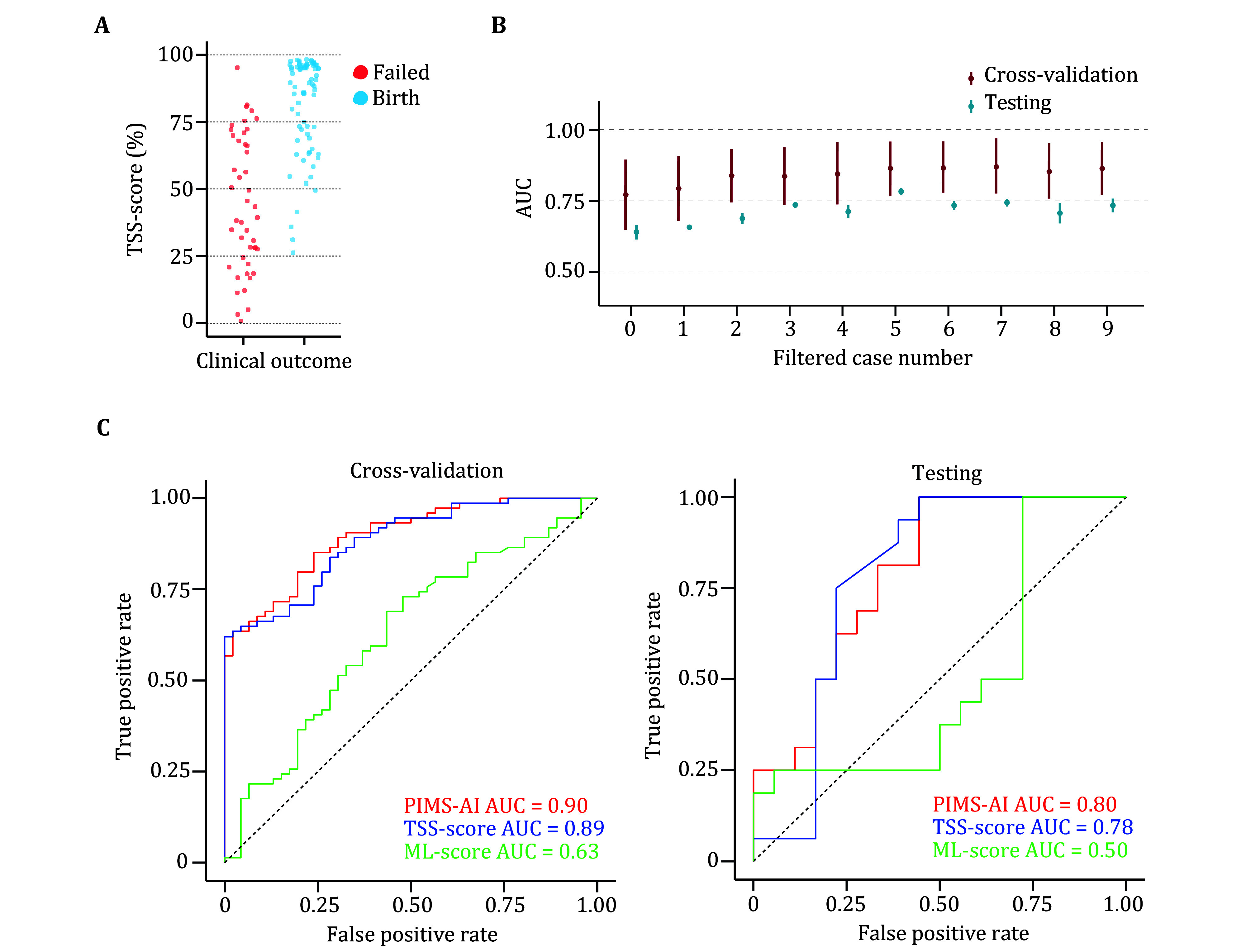
Data cleaning and embryo classification performance of PIMS-AI. **A** An example of noisy label identification, is a failed embryo with the highest TSS-score would be annotated as noise and excluded in the next iteration. **B** Learning-curve of TSS-score AUC relative to noise filtering. Error bars indicate 1 standard deviation. **C** AUC of ML-score, TSS-score and combined PIMS-AI model in cross-validation or independent testing. Results based on train set with the removal of 5 noise label

Inspired by the dramatic differences in promoter DNA methylation signatures, we developed an embryo classification model. For model training and validation, we divided the 160 transferred embryos from the PIMS clinical trial into a train set (*n* = 126) and a test set (*n* = 34). Embryos in the test set were from 16 patients who experienced multiple transfers and ultimately delivered a neonate. This train-test division facilitates our further simulation. Informative promoters were then selected based on methylation patterns in the train set. This allows a modeling with gradient boosting decision tree (GBDT), a machine learning model that is known for its excellent performance in a wide range of classification tasks (Greener *et al.* 2022; Zhang *et al.* 2017). In cross-validation and independent testing, this model makes a predicted birth probability, termed as TSS-score, for each embryo (Fig. 4A).

Our modeling procedure included an iterative data cleaning within the train set. We noticed that the conventional GBDT occasionally misjudges a small yet fixed portion of failed embryos (Fig. 4A). Besides these few exceptions, the model performed well and achieved an AUC greater than 0.75 in cross-validation with an unfiltered train set (Fig. 4B). However, the generalization performance is less ideal, with an AUC around 0.65 in independent testing when the model was trained by the unfiltered train set (Fig. 4B). These observations lead us to speculate that a few latent noises might be presented in our data label. The noisy label is a common problem in medical datasets and leads to suboptimal generalization performance, especially when having a limited sample size (Bootkrajang and Chaijaruwanich 2022; Karimi *et al.* 2020). A simple yet effective approach to overcome this problem is noise identification and exclusion (Song *et al.* 2023). The learning curve indicates an AUC increasement greater than 0.1 in both cross-validation and independent testing after a few rounds of cleaning (Fig. 4B). To avoid over-filtering, we made an early stop when only five cases (3.9% of total train set size) were removed. As we found the AUC of cross-validation remains close to 0.90 hereafter (Fig. 4B). These results indicate a reliable performance enhancement achieved by data cleaning.

To take advantage of performance on both whole genome and signature levels, we mixed the predicted scores of these two models together. TSS-scores are combined with linear transformed ML-score by a 1:2 ratio, resulting in the PIMS-AI model. This model achieved an AUC of 0.90 in cross-validation and 0.80 in independent testing (Fig. 4C). Meanwhile, as all embryos analyzed here are euploid, we considered that PGT-A cannot make any discrimination in these embryos (Gao *et al.* 2023). This suggests that PIMS-AI has a greater advantage than PGT-A in predicting embryo clinical outcomes.

### Accurate embryo discrimination and selection by PIMS-AI

**Figure 5:**
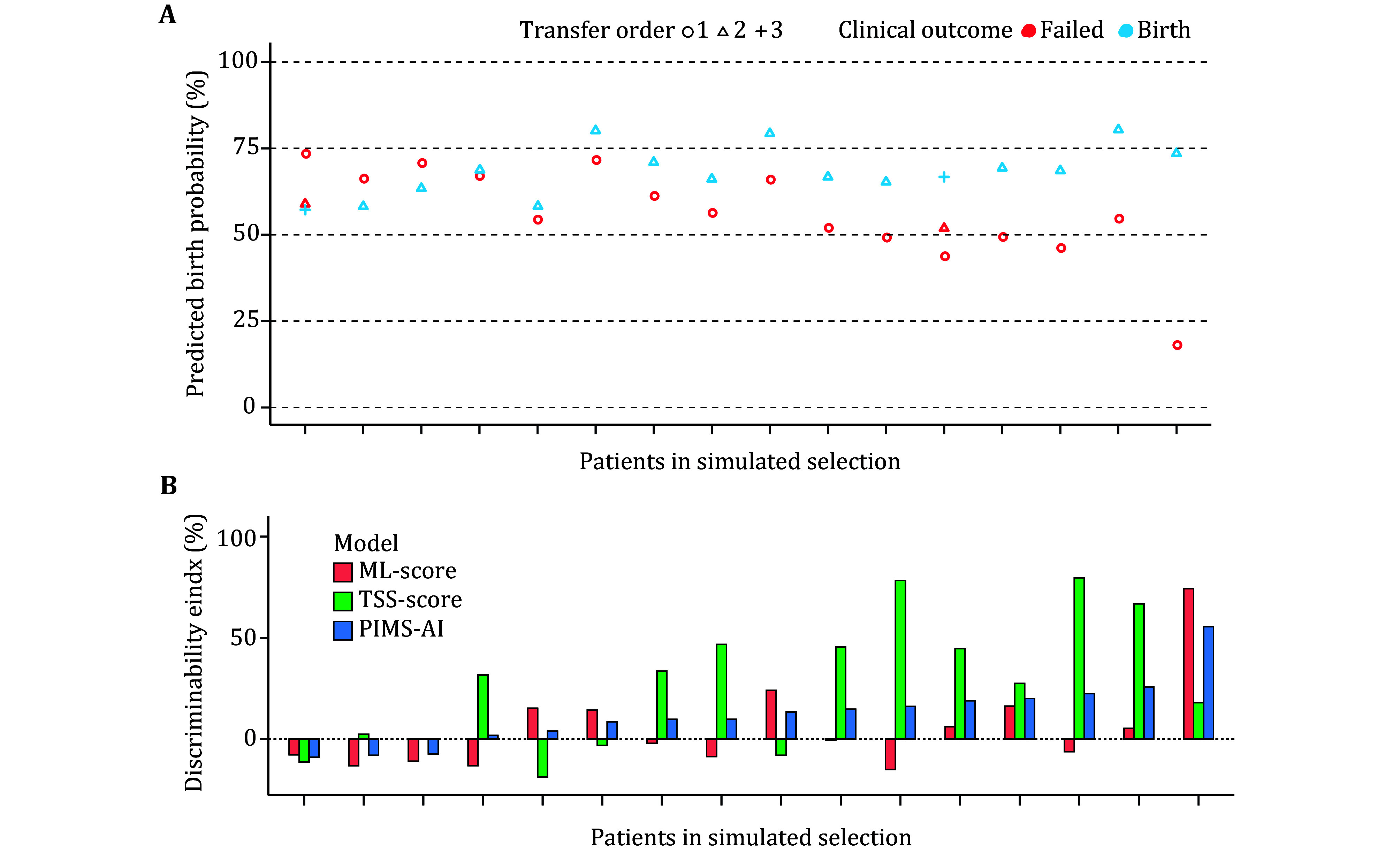
PIMS-AI performance in simulated embryo selection. **A** PIMS-AI prediction result of the embryo from each patient. Embryos from the same patient are plotted on the same column, with shape indicating their transfer order and color indicating their clinical outcome. **B** DI of ML-score, TSS-score and PIMS-AI in simulated selection for each patient. Patients are sorted in the same order as that in Panel A

To estimate the performance of PIMS-AI in clinical application, we conducted a simulated embryo selection using embryos from a test set. These embryos are from 16 patients, each has one birth embryo and one or two failed embryos. We found that PIMS-AI selected embryos correctly in 13 cases, and achieved an accuracy of 81% (Fig. 5A). Discriminability index (DI) was then employed to measure the ability of our model to distinguish embryos with different labels (Zou and Yuen 2010). Our results suggest PIMS-AI got an average DI of 12% for patients in simulated selections (Fig. 5B). TSS-score made a critical contribution to this performance, demonstrating the great potential of methylation signatures-based classification (Fig. 5B). We expect an even more powerful performance of PIMS-AI with further data collection. Importantly, as all analyzed embryos are euploid, conventional PGT-A could only make a random selection here. Compared with this scenario, our approach provides a significantly increased DI (*P* = 0.007). In conclusion, these results illustrate the great potential of PIMS-AI to take the place of PGT-A for future clinical application.

## DISCUSSION

Previous works have proposed using embryo whole genome DNA methylation level to indicate embryo clinical outcome (Gao *et al.* 2023; Li *et al.* 2017). In this study, we build a promising embryo classification and selection model based on detailed methylation signatures. Our findings further reinforced and illustrated the clinical potential of PIMS.

Although being globally offered, the clinical efficacy of PGT-A is still under debate (Gleicher *et al.* 2022). PGT-A mainly brings benefits to women with advanced maternal age, because the risk of producing an aneuploid embryo significantly increased with higher maternal age (Niederberger *et al.* 2018). In contrast, we found embryo methylome heterogeneity existed in women of all ages. Furthermore, our model significantly outperformed the current method of PGT-A in embryo classification and selection. Based on these results, we expect PIMS to improve the ART success rate for the overall populations.

Epimutations disturb embryo development and cause pregnancy failure. We revealed a number of epimutations and their related pathways. A robust embryo selection model was proposed by analyzing these epimutations. Our previous work indicates screening epimutations on important regulatory regions might decrease the birth defect rate (Gao *et al.* 2023). In a future clinical implementation, we expect that birth defects could also be predicted by PIMS with a comprehensive analysis of epimutations.

Taken together, this study established an AI-based embryo selection model that utilizes methylation profiles. Results suggest our model has a robust performance in accurately selecting viable embryos for ART patients. Model performance in simulations indicates a significant improvement in clinical outcomes with PIMS application in the future.

## MATERIALS AND METHODS

### Data processing and annotation

Methylation profile and clinical records were acquired from the PIMS clinical trial, and methylation level estimation was also performed using a method similar to this clinical study (Gao *et al.* 2023). Briefly, the mean methylation level of all sequencing covered CpG was used to represent whole genome DNA methylation level. To estimate DNA methylation level in promoters, gene reference on hg19 was downloaded from the UCSC table browser. The gene promoter region was defined as 1000 bp upstream and downstream of TSS. The average DNA methylation level of sequencing covered CpG in a promoter was used to represent the DNA methylation level of the corresponding promoter in the corresponding embryo. To filter low-coverage regions, promoters with less than 30 times of total CpG coverage were removed.

In downstream analysis, embryos were annotated as "Birth" if resulted in a live birth, and annotated as "Failed" if resulted in pregnancy failure or pregnancy loss.

### Methylation level score and linear model

For a given embryo *i*, the methylation level score was calculated by the following equation:

1 $ {ML\;score}_{i}=abs{(ML}_{i}- 0.26), $

where *ML*_*i*_ represents the whole genome DNA methylation level of corresponding embryo *i*, presented in decimals. ML-scores and clinical outcomes of all 160 embryos were next applied in fitting a generalized linear model using function "glm" in R version 4.1.2, with "formula" set as "clinical outcome" ~ "ML-score" and "family" set as "binomial". In model fitting, the embryo clinical outcome was assigned as 1 if they produced a live birth, and assigned as 0 if they failed to produce a live birth.

To illustrate the fitting result, the simulated ML-score ranged from 0 to 0.3 with an interval of 0.01 was used. The fitted model returns a predicted birth probability and standard error for each simulated score. These results were presented in Fig. 1B, except for values that were less than 0.

### Estimate PIMS clinical efficiency with a linear model

We first anticipate 0.03 as the threshold for a noticeable difference in ML-score to determine significant embryo quality difference. Next, we select patients that have two or more euploid embryos before their first embryo selection to perform downstream analysis. Their first embryo selection was retrospectively analyzed based on the following method.

For a given patient *j* with euploid embryos *j,i*_*0*_ to *j,i*_*n*_ , we calculate the index of embryo heterogeneity (H-index) based on the following equation:

2 $ {H}_{j}=\mathrm{max}\left({ML\;score}_{j,i}\right)-\mathrm{min}\left({ML\;score}_{j,i}\right), $

where *H* represents embryo heterogeneity, the distance between the highest and the lowest ML-score in the available embryos was applied in the calculation. The index of selection status (S-index) was calculated for patient *j* based on the following equation:

3 $ {S}_{j}={ML\;score}_{j,\mathrm{s}\mathrm{e}\mathrm{l}\mathrm{e}\mathrm{c}\mathrm{t}\mathrm{e}\mathrm{d}}-\mathrm{m}\mathrm{i}\mathrm{n}\left({ML\;score}_{j,i}\right) , $

where *S* represents the selection status, the distance between ML-score of the selected embryo and the lowest ML-score in all of the embryos (including the selected one) were applied in the calculation.

Patients were then categorized into three groups according to their *H* and *S* value: patients with *H* value less than 0.03 as having embryos with similar quality (Fig. 2B); patients with *H* greater than 0.03 while *S* less than 0.03 as selected an embryo with superior quality (Fig. 2C); patients with *H* and *S* both greater than 0.03 as selected an embryo with inferior quality (Fig. 2D).

For each group, the clinical results of their embryo transfer were summarized and compared using Fisher’s exact test.

### Detection of methylation mutation in promoters

To find DNA methylation mutation in promoters, we first filtered promoters with low data coverage. Promoters covered by less than 10% of the sample in either group ("Birth" or "Failed") were removed from further analysis. Next, the mean and standard deviation of the birth group on each promoter were calculated. Subsequently, we detect epimutations using the following equation:

4 $ \left\{
\begin{aligned} &
{AM}_{m,n}=abs({ML}_{m,n}-{ML}_{\mathrm{b}\mathrm{i}\mathrm{r}\mathrm{t}\mathrm{h},n})\\& {RM}_{m,n}=\frac{{AM}_{m,n}}{{S.D.}_{\mathrm{b}\mathrm{i}\mathrm{r}\mathrm{t}\mathrm{h},n}}
\end{aligned}\right. , $

where *AM*_*m,n*_ represents the absolute mutation level of embryo *m* on promoter *n*, *ML*_*m,n*_ represents the DNA methylation level of embryo *m* on promoter *n*, *ML*_birth*,n*_ represents the mean DNA methylation level of all birth embryos on promoter *n*, *RM*_*m,n*_ represents the relative mutation level of embryo *m* on promoter *n*, *S.D.*_birth,*n*_ represents the DNA methylation level standard deviation of all birth embryos on promoter *n*. We defined the epimutation identification cutoff as AM greater than 0.1 and RM greater than 3.

For each embryo, we calculate their individual epimutation frequency on all promoters using the count ratio of detected epimutation against all sequencing covered promoters. For the birth and failed groups, we calculate their group epimutation frequency on each promoter using the count ratio of the epimutation sample against all effectively covered samples.

To find epimutations related pathways, we extract genes whose promoters have higher epimutation frequency in the failed group. Gene ontology analysis was performed on geneontology.org with enrichment significance calculated by a binomial test.

### Machine-learning based TSS-score prediction

To build a prediction model, we first extract informative features from all promoters by applying ANOVA and the Wilcox test. Promoters with a difference significance less than 0.05 in either test are included in model fitting. Statistical and model fitting analysis were performed using Jupyter Notebook V6.4.7, python V3.8.10 and classification model "gradient boosting decision tree" in Scikit-learn V1.0.2 with default parameters.

For the prediction of a given embryo using the same train set, average prediction scores of multiple times (*N* > 10) of model fitting were used as the final prediction. This is because we found our limited sample size results in slightly different scoring at each different time of fitting, as the parameter "random_state" was never fixed in our analysis.

To iteratively remove noise labelled sample by cross-validation, we calculate the probability for a given embryo *i* of being noisy using the following equation:

5 $ {N}_{i}=abs\left({R}_{i}-{P}_{i}\right), $

where *N* represents the noise probability, *R* represents its clinical result (0 for failed and 1 for birth), and *P* represents its prediction score in the corresponding iteration. A failed embryo with maximum noise probability would be removed after each iteration.

In order to avoid bias induced by sample distribution in cross-validation. Multiple times (*N* > 10) of 5-fold cross-validation with different sample distributions were performed in each iteration.

### Simulate clinical application by simulated selection

For patients in the independent test set, the discriminability index was calculated for each of them using the following equation:

6 $ {DI}_{j}={P}_{j,\mathrm{b}\mathrm{i}\mathrm{r}\mathrm{t}\mathrm{h}}-{P}_{j,\mathrm{f}\mathrm{a}\mathrm{i}\mathrm{l}\mathrm{e}\mathrm{d}} , $

where *DI*_*j*_ represents the discriminability index for patient *j*, *P*_*j,*birth_ represents the predicted score for the birth embryo of patient *j*, *P*_*j,*failed_ represents the average predicted score for the failed embryo of patient *j*.

To estimate DI improvements brought by PIMS-AI, DIs of the PIMS-AI model in simulated selection were applied in a one-sample student *t*-test.

### Abbreviation

**Table:** 

AI	Artificial intelligence
ART	Assisted reproductive technology
AUC	Area under the curve
DI	Discriminability index
GBDT	Gradient boosting decision tree
GLM	Generalized linear model
PGS	Preimplantation genetic screening
PGT-A	Preimplantation genetic testing for aneuploidy
PIMS	Preimplantation DNA methylation screening
TSS	Transcription start site

## Conflict of interest

Jianhong Zhan, Chuangqi Chen, Na Zhang, Shuhuai Zhong, Jiaming Wang, Jinzhou Hu and Jiang Liu declare that they have no conflict of interest.
